# Quality Improvement in the Practice of Blood Transfusions in Transcatheter Aortic Valve Implantation Procedures

**DOI:** 10.7759/cureus.86348

**Published:** 2025-06-19

**Authors:** Adeogo A Olusan, Garima Gupta, Monica Rajendran, Shazaan Nadeem, Madiha Iqbal, Shazia Hussain, Amerjeet Banning, Raj Rajendra, Elved Roberts, Jan Kovac

**Affiliations:** 1 Cardiology, University Hospitals of Leicester NHS Trust, Leicester, GBR

**Keywords:** blood cross-matching, cost-effectiveness, non-surgical tavi, packed red cell transfusion, quality improvement, transcatheter aortic valve implantation, transfemoral access

## Abstract

Background

Transcatheter aortic valve implantation (TAVI) is a standard procedure for symptomatic severe aortic stenosis and is especially favored in patients with a high surgical risk. Although TAVI is minimally invasive, blood transfusion remains a relevant postoperative concern. Glenfield Hospital routinely cross-matched four units of packed red cells (PRCs) for every TAVI patient. This practice put significant strain on transfusion services and hospital resources with the increased volume of TAVIs performed, from 182 cases in 2021 to 543 in 2024. Changes in data regarding blood transfusion demands allowed for changing the practice to cross-matching only two units of PRCs for non-surgical transfemoral TAVI patients starting June 2024. This study aimed to assess the safety and cost implications before and after implementing the revised blood cross-matching protocols for non-surgical transfemoral TAVI patients.

Methodology

A retrospective study was performed among all TAVI cases at Glenfield Hospital from June 1 to December 31, 2024. The study used the National Institute for Cardiovascular Outcomes Research TAVI database. Patients were split into different groups according to approach (non-surgical transfemoral access vs. surgical access). The primary aim was measurement of the transfusion rate and the number of units transfused after the TAVI procedure, and the secondary aim was to assess the safety and cost implications before and after implementing the revised blood cross-matching protocols for non-surgical transfemoral TAVI patients.

Results

Out of 312 patients (mean age = 81 ± 6 years; 39% female), 94% received non-surgical transfemoral TAVI. Only four (1.28%) patients required PRC transfusion. Only one (0.32%) patient required more than two units following vascular closure device failure, requiring surgical repair.

Conclusions

This single-center study offers evidence to justify the modified practice of limiting cross-matching of blood to two PRC units, or doing group and save only, in non-surgical transfemoral TAVI patients. The practice is safe as well as cost-effective and leads to significant cost savings without compromising patient care. However, further multicenter, prospective studies with long-term follow-up are justified to replicate these findings and establish broader implications for patient care.

## Introduction

Severe aortic stenosis is a life-threatening disease that is more prevalent in the elderly population. Over the years, transcatheter aortic valve implantation (TAVI) has proven to be a safe and effective alternative to surgical valve replacement in more than a decade’s worth of experience, particularly for high-risk surgical patients [1]. As modern technology and techniques have evolved, TAVI is now routinely performed with only local anesthetics via the femoral artery (minimally invasive surgery). Blood transfusion after TAVI, although safe, remains a major concern because of its consequences and impact on healthcare expenditures. Published data have shown that the rate of packed red cell (PRC) transfusion following TAVI is between 16% and 39% [2-4]. Transfusion practices differ widely from one institution to another, frequently in the absence of contemporary baseline evidence available in the literature [5,6]. Glenfield Hospital is a TAVI benchmarking hospital, which for years has had a TAVI policy of cross-matching four units of PRCs per TAVI patient irrespective of individual bleeding risk. This becomes logistically and financially unsustainable in the face of tripling TAVI procedures between 2021 and 2024 [7]. An initial study in May 2024 from Glenfield Hospital, Leicester, presented at PCR London Valve conference in November 2024 found that the rate of PRC transfusion spanning over 11 years was approximately 5.9% (111/1878), and even more astonishingly 1.38% (26/1878) in excess of two units in non-surgical transfemoral TAVIs [8]. With this information, we adopted a protocol aimed at reducing cross-matching to two units for non-surgical TAVI patients in June 2024. This quality improvement project (QIP) examines the transfusion rate and the number of units transfused after the TAVI procedure, and the safety and cost implications before and after implementing revised blood cross-matching protocols for non-surgical transfemoral TAVI patients.

## Materials and methods

This retrospective study was conducted among consecutive patients who underwent TAVI at Glenfield Hospital, Leicester, UK, between June 1 and December 31, 2024. As with all interventional procedures in the UK, all data are recorded into the National Institute for Cardiovascular Outcomes Research (NICOR) registry. We extracted these data from the NICOR TAVI database, which included all patients who underwent TAVI at Glenfield Hospital during the study period, along with patient and procedural characteristics. Patients with the presence of red cell antibodies and surgical access to TAVI routes (subclavian, transapical, or direct aortic) were managed under a protocol of cross-matching four PRC units and were thus excluded from this analysis.

The data from the NICOR database were contemporary and complete, and no patient received more than one TAVI valve/procedure during the study period. We obtained audit registration and approval for this QIP on December 16, 2024.

In terms of definition, non-surgical transfemoral TAVIs are performed via the femoral artery without an arterial cut-down, i.e., percutaneous access, whereas surgical access TAVIs are performed using arterial cut-down for femoral artery and subclavian artery cut-down, transapical, transcaval, and direct aorta access. For transfusion requirement, there must be a need for at least one unit of PRC transfusion during or after the procedure is performed.

Non-surgical transfemoral TAVI patients had their cross-matching altered to two PRC units from June 2024. However, from November 2024, our blood transfusion department routinely stopped preoperative cross-matching for non-surgical transfemoral TAVIs unless red cell antibodies were present. This followed a separate internal audit carried out by the transfusion department unrelated to our QIP. Nevertheless, the hospital protocol remains in place for group and save.

The new protocol followed a formal audit/governance pathway and obtained multidisciplinary consensus for its adoption following data presentation at the Glenfield Hospital bi-monthly cardiology audit meeting on May 30, 2024. The findings of this QIP were presented at the audit meeting on February 27, 2025, with consensus for it to be promoted to a Cost Improvement Programme.

Glenfield Hospital’s standard practice regarding anticoagulation and antithrombotic therapy is that warfarin is held at least three days before TAVI procedures, Novel oral anticoagulants (NOACs) are held 48 hours before TAVI procedures, and both Warfarin and NOAC are restarted the next day after TAVI. Those on warfarin may or may not require low-molecular-weight heparin (LMWH) bridging to therapeutic international normalized ratio levels before discontinuation of LMWH, depending on the indication. However, patients on antiplatelet therapy do not require discontinuation of these before TAVI.

This study was adequately powered. The sample size for this QIP was 246, considering a population proportion of 20%, an error margin of 5%, and a confidence interval of 95%. Statistical analyses were performed using StataNow 19 BE. Continuous variables are presented as mean ± standard deviation or median (interquartile range), depending on distribution. Categorical variables are expressed as percentages. Z-score tests were used to test for statistical significance by comparing the proportion of blood transfusion rates between the first and second study. A p-value ≤0.05 indicated statistical significance.

## Results

A total of 312 patients received TAVI during the six-month study period. Baseline demographic and clinical characteristics are shown in Table 1. The mean age was 81 ± 6 years, and 39% (122/312) were female. Overall, 28% (89/312) have a history of diabetes mellitus, 9% (29/312) a history of stroke, 7% (22/312) a history of transient ischemic attack, 27% (85/312) a history of atrial fibrillation/flutter, and 5% (17/312) had severely impaired left ventricular function (ejection fraction: <35%). As far as procedural characteristics are concerned, the majority of the procedures were performed under local anesthesia, 94% (294/312) of TAVIs were non-surgical transfemoral access TAVIs, 52% (162/312) of patients received self-expanding valves versus 48% (150/312) balloon-expanding valves, 29% (91/312) required pre-dilation balloon aortic valvuloplasty, and 1% (3/312) required post-dilation following valve implantation (Table 2). Most patients required a 14-Fr sheath for their TAVI procedure, whereas those with larger valves, for example, 29 mm Edwards Ultra/RESILIA, 34 mm Evolut FX/FX+, 26 - 27.5 - 29 - 30.5 mm - 32XL MyVal Octacor, required an 18-Fr sheath.

**Table 1:** Baseline characteristics of the patients. BMI: body mass index; MDT: multi-disciplinary team; COPD: chronic obstructive pulmonary disease; ILD: interstitial lung disease; TIA: transient ischemic attack; MI: myocardial infarction; IQR: interquartile range; AF: atrial fibrillation; TAVI: transcatheter aortic valve implantation; LV: left ventricular; EF: ejection fraction

Patient characteristics	Number (%), n = 312
Age	81 ± 6
Female	122 (39%)
Ethnicity	
Caucasian	297 (95.5)
Black	1 (0.5)
Asian	13 (4)
Procedure urgency	
Elective	241 (77)
Urgent	73 (23)
Body mass index, BMI	28 ± 5
Heart team MDT performed	312 (100)
Diabetes	89 (28)
Smoking status	
Current smoker	14 (4)
Ex-smoker	176 (56)
Never smoked	122 (39)
Pulmonary disease	
Asthma	28 (9)
COPD	44 (14)
ILD	20 (6)
Neurological disease	
Stroke	29 (9)
TIA	22 (7)
Previous MI	29 (9)
Creatinine, mmol/L (IQR)	92 (77, 116)
Heart rhythm	
Sinus	194 (62)
AF/flutter	85 (27)
Paced	30 (10)
Unknown	3 (1)
Aortic valve pathology	
Stenosis	294 (94)
Regurgitation	6 (2)
Mixed	12 (4)
Aortic valve etiology	
Degenerative	284 (91)
Bioprosthetic	25 (7.5)
Rheumatic	2 (1)
Congenital	1 (0.5)
Pre-TAVI echo parameters	
Peak gradient, mmHg	70 ± 24
Mean gradient, mmHg	41 ± 16
Valve area, cm²	0.78 ± 0.29
LV systolic function	
Preserved, EF > 55%	240 (77)
Impaired, EF 35–49%)	55 (18)
Poor, EF < 35%	17 (5)

**Table 2:** Procedural characteristics of the patients. TAVI: transcatheter aortic valve implantation; NOAC: novel oral anticoagulants

Procedural characteristics	Number (%), n = 312
Delivery approach	
Transfemoral	294 (94)
Femoral (surgical cut-down)	1 (0.5)
Subclavian	10 (3)
Direct aortic	6 (2)
Trans apical	1 (0.5)
Procedure time, minutes	66 ± 25
Anesthesia	
General	18 (6)
Local	294 (94)
Valve platform	
Medtronic, Evolut FX/FX+	112 (36)
Edwards, S3 Ultra/RESILIA	97 (31)
Meril MyVal, Octacor	53 (17)
Boston, Acurate Neo 2	42 (13)
Biosensor, Allegra	8 (3)
Valve size	
20–23	85 (27)
24–27	136 (44)
29–34	91 (29)
Balloon pre-dilation prior to TAVI	91 (29)
Post-dilation after TAVI	3 (1)
Vascular closure technique	
Device closure	291 (93)
Surgical closure (planned)	18 (6)
Surgical closure (bailout)	3 (1)
Post-TAVI echo parameters	
Peak gradient, mmHg	17 ± 9
Mean gradient, mmHg	8 ± 5
Valve area, cm²	2.02 ± 0.55
Blood transfusion after TAVI	
None	306 (98.71)
1 unit	1 (0.32)
2 units	2 (0.65)
5 units	1 (0.32)
Permanent pacemaker implantation	
None	251 (80)
After TAVI	28 (9)
Prophylactic before TAVI	33 (11)
Antiplatelet therapy on discharge	
Aspirin only	126 (40)
Aspirin + clopidogrel	14 (4)
Aspirin + ticagrelor	1 (0.5)
Clopidogrel only	48 (15)
Antithrombotic therapy on discharge	
Warfarin	11 (4)
NOAC	117 (38)

**Figure 1:**
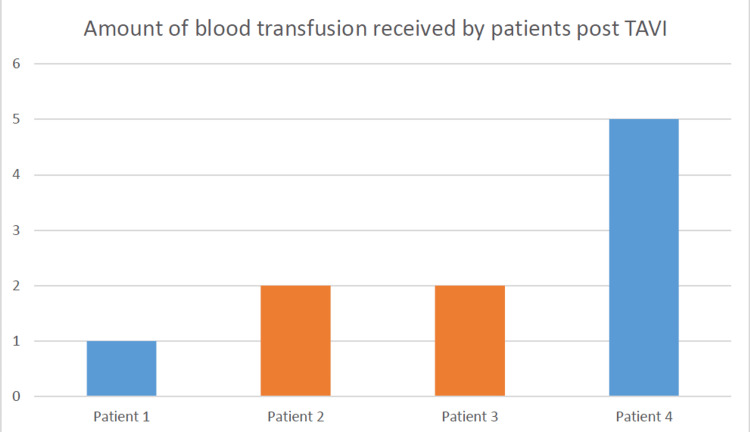
Blood transfusions received by patients post TAVI. TAVI: transcatheter aortic valve implantation

The mean procedure time was 66 ± 25 minutes, and 93% (291/312) required vascular closure device deployment using Perclose Proglide ± Angioseal. Overall, four (1.28%) patients required PRC transfusion in this study, with only one (0.32%) patient requiring more than two units of PRC transfusion, due to emergency repair of the femoral artery following failure of the vascular closure device (Figure 1). Regarding cost, based on figures from 2012, the estimated cost of cross-matching two units for 312 patients was £137,070.96 as against £274,141.92 for four units of PRC cross-match. Hence, we saved an equivalent amount of unnecessary costs (Table 3). This is an estimated cost based on the current inflation rate of 3%.

**Table 3:** Cost saving scenario and outcome.

Scenario, n = 312	Total cost (£)	Cost avoided (£)
Cross-matching two units per patient	137,070.96	137,070.96
Cross-matching four units per patient	274,141.92	0

## Discussion

This QIP demonstrated that the reduction of preoperative cross-matching to two units, or even abolition in favor of group and save, is a safe strategy for non-surgical transfemoral TAVI patients. The evidence supports that the vast majority of these patients do not require blood transfusion, and those who require it need no more than two units. Our data showed that the rate of PRC transfusion following TAVI was 1.28%, which is significantly lower than that published in the literature [2-4]. Various factors can influence the rate of PRC transfusion following TAVI, such as addressing anemia before TAVI which helps mitigate the need for post-TAVI PRC transfusion [9], adjusting antithrombotic medications, improving sheath profiles (less bleeding with small sheaths than larger sheaths), improving delivery catheter systems, and the use of vascular closure devices. Moreover, greater experience with the TAVI procedure and vascular closure techniques can also lead to lower transfusion rates [2].

As a result of the increased volume of our yearly TAVI procedures at Glenfield Hospital from 182 in 2021 to almost a three-fold increase (543) in 2024, this translates into a better experience, fewer complications, and lower transfusion rates [7]. This is similar to findings from a study from the United States, which showed a significant reduction in the PRC transfusion rate during the first quarter of 2012 and the third quarter of 2015 [10]. The initial study from May 2024 showed that 5.9% (111/1,878) of patients required PRC transfusion between January 2013 and 2024, with 1.38% (26/1878) requiring more than two units of PRC transfusion [8]. In comparison, this study showed that 1.28% (4/312) of patients required PRC transfusion (i.e., 5.9% vs. 1.28%, p = 0.0007, 95% confidence interval (CI) = 1.46-7.74%) and 0.32% (1/312) requiring more than two units of PRC transfusion (i.e., 1.38% vs. 0.32%, p = 0.107, 95% CI = -0.307-0.947%), though this was clinically relevant and acceptable in those requiring more than two units but not statistically significant. Similarly, in the early years of TAVI, vascular access was closed surgically; however, with the use of vascular closure devices, this translates into reduced bleeding complications and transfusion rate.

The economic impact of this protocol change is significant. Based on the 2024 NHS Blood and Transplant tariffs [11-13], the cost of cross-matching two units of PRC was approximately £294.60 in 2012, which would be higher now after considering inflation [5]. Assuming an average annual healthcare inflation rate in the UK is approximately 3% using the following formula: future cost = original cost × (1 + r)^n^, where r = 0.03 (i.e., 3.0%) and n = 13 years from 2012 to 2025, this translates into future cost = £294.60 × (1 + 0.03)^13^ => £294.60 x 1.491 = £439.33 for two units in 2025. Prevention of unnecessary cross-matching in 90% of TAVI cases a week (approximately 13 out of 15) would save thousands of pounds a week in transfusion services, as well as reduce workload pressure. The findings are in agreement with reports from other surgical specialties demonstrating the safety and financial advantage of personalized blood management strategies [5,6].

The authors acknowledge that the volume-outcome relationship is plausible; however, other concurrent changes may have also contributed to the low transfusion rate, for example, better patient selection and newer device iterations. Furthermore, most of our cross-matching is done electronically, which takes approximately five minutes. Hence, in cases of emergency bleeding, O-negative blood can initially be transfused pending the results of cross-matching. Patients who have antibodies are routinely cross-matched along with surgical access TAVI patients so that there are available blood units for transfusion in cases of emergency. This study only addresses patients without antibodies and non-surgical access TAVIs.

This study has several strengths. First, it is derived from real data of a large TAVI patient population, thus providing robust evidence to guide the revised transfusion protocol. Second, by reducing units of cross-matched blood, the intervention alleviates pressure on hospital resources, following best practices in patient blood management. Third, the low transfusion rate of 1.28% for one to two units of PRC transfusion, with 0.32% requiring more than two units, highlights the safety of the new protocol. Fourth, this study highlights the cost-effectiveness of the new protocol, demonstrating a balance between cost savings and patient safety. Finally, our findings suggest that quality improvement efforts such as this can be implemented in other centers, allowing widespread adoption of streamlined transfusion practices.

Nevertheless, there are a few limitations of this study. This retrospective study design lacked a control group or an incomplete adjustment for confounders. This single-center study may not be generalizable to other centers with different patient populations, resources, or TAVI experience. Unmeasured variables, such as TAVI technique and skill or patient comorbidities, may influence transfusion requirements and complicate the interpretation of results. The study solely examines the transfusion practice and does not evaluate other aspects of patient care or TAVI-related outcomes.

In summary, this QIP from a single center demonstrates the feasibility and benefit of revising transfusion protocols in TAVI patients based on short-term data, pending validation in a broader population. These findings are most relevant to similar centers performing high-volume, non-surgical transfemoral TAVIs. However, further multicenter, prospective studies with long-term follow-up are justified to replicate these findings and establish broader implications for patient care.

## Conclusions

This single-center study offers evidence to justify the modified practice of limiting cross-matching of blood to two PRC units, or doing group and save only, in non-surgical transfemoral TAVI patients. The practice is safe as well as cost-effective and leads to significant cost savings without compromising patient care. However, further multicenter, prospective studies with long-term follow-up are needed to replicate these findings and establish broader implications for patient care.
